# Report Made in the Name of the Committee on Mineral Waters for 1837

**Published:** 1839-06-15

**Authors:** Ph. Patissier


### MEDICAL EXAMINER.

DEVOTED TO MEDICINE, SURGERY, AND THE COLLATERAL SCIENCES.

No. 24.] PHILADELPHIA, SATURDAY, JUNE 15, 1839. [Vol. II.

*Report made in the name of the Committee on Mineral Waters for* 1837. By M. Ph. Patissier.

(Continued from page 326.)

Chap. 11.—*On the manner in which Mineral Waters act.*

There are two things to be particularly remarked in the action of mineral waters taken at their springs. 1st. They act as a hygienic agent; this manner of acting is common to all, and powerfully assists their medicinal properties. 2d. They act by means of their constituent principles, which impress upon the organization changes more or less pronounced. It appears from an examination of the clinical facts reported by the inspecting physicians, that mineral waters employed in drinking, in baths, in douches, and in vapour baths, always stimulate internally or externally with more or less force. They act principally, then, by stimulating in different degrees, by promoting in the organs more abuudant secretions and excretions, and, in certain cases, by bringing back to their normal state those which exercise these functions with too much energy; but to be useful, this stimulation should be maintained within just limits; if it is slow and gradual, it relieves and cures old complaints; if too strong, it exasperates them and revives latent sources of irritation.

This stimulation, which Borden compares to that of coffee, and whose intensity bears a relation to the proportion of saline and gaseous matters contained in the water, to the temperature of the baths, and to the nervous susceptibility of the patient, is apparent after a few days of treatment, by general lassitude, depression of strength, wakefulness, increase of pains, inflammation of wounds and issues, and by a febrile movement; in a word, it brings chronic diseases to a stage momentarily acute. One of the results, then, of the use of mineral waters, is to aggravate, for a brief period,* chronic affections; this constitutes a very frequent transition from disease to cure. This exacerbation is sometimes so acute, that the inspecting physician is obliged to suspend, for a time, the use of the water, and to prescribe cooling drinks, emollient baths, and even bloodletting. Nor is it rare for patients to recover, under the influence of a mineral water, their appetite and cheerfulness, and, soon afterwards, to see their old sufferings return with a new intensity; it is thus that, during the use of mineral baths, rheumatic pains are revived, or are exasperated, phenomena which are almost always the prelude of their cessation. It is then a very frequent effect of the treatment to revive allayed pains, or to exacerbate those already existing, for their complete removal afterwards. It is very important that patients should be informed of this action of the waters, in order that they may not allow themselves to be discouraged, and thus lose the benefits of a treatment, commenced, in appearance, under unfavourable auspices.

Mineral waters act principally upon two great surfaces; in drinking, upon the gastro-intestinal mucous membrane; in baths, douches, and vapour baths, upon the skin; they excite there two membranes, which, in their turn, act upon the other organs connected with them, by numerous sympathies, increase the activity of their functions, and modify their vitality. Conveyed in the act of drinking, into the primæ viæ, absorbed and carried into the most minute ramifications of the vascular system, the water penetrates every portion of the economy, and establishes in the diseased organ a functional labour, of greater or less activity; under its influence the strength is invigorated; the circulation, before languid, resumes its activity; warmth is recalled into the parts which it had abandoned; and the whole body is animated with a new movement, or, in the words of Borden, is *new mounted throughout.*

Mineral waters are, some purgative, some diuretic, some diaphoretic, according to the nature of the principles which compose them, the particular state of the individual who uses them, the quantity which he takes, the manner in which they are administered, and the different circumstances which precede, or which accompany their employment.

The temperature of mineral baths has also a great influence upon the stimulation of the economy; thus the same bath which might be emollient at 35° cent., (95° Far.,) would become powerfully stimulating at 39° to 41°, ($102\frac{4}{5}$° to $105\frac{1}{5}$°,) and is a powerful irritant at 42° to 45°, ($107\frac{3}{5}$° to 113°,) a temperature which can be supported only during a few moments. It is then essential to watch with care the graduation of the temperature of baths; it is in this that consists, in part, the secret of the surprising cures effected sometimes in thermal establishments. We must not think, however, that the stimulation resulting from mineral baths depends solely upon the degree of their temperature; the quantity, and greater or less energy of the mineral principles which they contain, contribute also to develope it. We know, indeed, that the sulphurous ingredients, that carbonic acid, the alkaline carbonates, the ferruginous salts, and other saline matters, are not

* At Greoulx, 20 patients out of 301 experienced an aggravation of their diseases, and at Plombieres, 18 out 107 became worse, at least for a short time.

without action upon the skin; experience teaches us that the more active principles the warm mineral waters contain, the shorter time the patient should remain in the bath. Thus the use of the sulphur baths of Bourbonne, of Balaruc, of Mont d'Or, of Vichy, &c., taken even at a moderate temperature, ought not to be continued more than a half hour or an hour at a time, lest there be too great an excitement of the nervous system,* whilst that of the warm baths of Nevis, Luxeuil, Bains, Plombieres, Bagneres de Bigorre, Bourbon, Lancy, &c., which are rather deficient in ponderable principles, and, consequently, little exciting, can be prolonged with advantage to the patients for three or four hours. The proof of the tonic effects of mineral bathing is, that their use, although repeated every day for a month, instead of enfeebling the patients, as baths of common water do, strengthens them, and imparts a new energy to all their functions, which effect depends, without doubt, upon the constituent elements of the water upon their degree of temperature, and perhaps upon the electric fluid which they contain.

We will say nothing of the stimulating effects of douches and vapour-baths, inasmuch as their action is well known to all physicians. As to sea bathing, it excites the skin so strongly, either by the saline principles of the water, or by the percussion of the waves, that after being continued for a few days, the patients complain of itching and redness of the skin, and of eruptions, similar to those of measles or scarlatina.

The stimulating effects of mineral waters are, as we see, of high practical importance; the physicians of preceding ages, without doubt, fixed the *seasons*, that is to say the space of time during which patients should remain at watering places, in consequence of their observations on this head. The duration of the mineral treatment is ordinarily from twenty to thirty days. Although many chronic diseases cannot be cured in so short a time, it is no less true that if the water has not produced the desired effect at its expiration, it is necessary in general to discontinue its use, inasmuch as its continuation will be more injurious than useful; in like manner, if after this period the patients do not find themselves cured or partially relieved, they should suspend the use of water, that the benefit obtained from it may not be undone. Indeed, as mineral waters have the effect of bringing chronic diseases to an acute stage, there is a degree of stimulation which cannot be passed without danger. If the patients persist in not observing the prescribed limits in their use of the water, there finally supervenes a general uneasiness, thirst, bad taste, dryness of the skin, fever; in a word, all those symptoms which announce that excitement has passed to inflammation. Custom has then with reason established the *seasons*, which ought to be the shorter, as the action of the water is more energetic. The arguments against a prolonged treatment, may likewise be urged against taking mineral waters a short time after having discontinued their use, or against making a second *season*. However, when the water is mild and little exciting, it may be taken again to advantage after the lapse of a fortnight.

The stimulation from the use of mineral waters accounts also for their consecutive or slow effects. If the salutary action of the mineral liquid is sometimes felt during its use, it happens more frequently that it manifests itself, as we have before said, some time after. We can readily conceive that an excitement which has pervaded all the tissues, must remain a longer time in the diseased organ, and that it must be calmed before the beneficial effects derived from the water become apparent. For this purpose it is necessary that the patients, after quitting the watering place, should continue for a month or two the diet which was prescribed during their use of the water, and that they should abstain from all active remedial treatment. Hence, in order to know what may be the power of mineral waters, it is often much better to interrogate patients who have stopped taking them, than those who still continue to take them.

The reflections which we have submitted to you, gentlemen, prove to you that in the excitement of the whole economy resides one of the great medicating forces of mineral waters with the diseased individual; the same excitement is also manifested, though in a manner less apparent, with the healthy individual; in a state of health, mineral waters, drank in small quantities, increase, in general, our appetites, and give a greater activity to our functions; administered in baths at the temperature of 35° cent. (95° Fah.) they impart a new vigour to muscular action; but the question may be asked, if mineral waters may be used with impunity in drinking and bathing in a state of health, how can they do injury in a state of disease?* This can be explained without difficulty: in the first case they invigorate equally all the systems of the economy; whereas, in the second case, they communicate an additional excitement to the diseased organ, which when too strong becomes dangerous. Under these circumstances, mineral waters have similar effects to those of wine and coffee, which, as we know, are powerful stimulants. We can without inconvenience, and even with advantage in a state of health, make a moderate use of these liquors, which become injurious in a state of disease.

It appears then, from the above, that the physician in prescribing the mineral water, and

* It is for the same reason that sea-baths ought to be of short duration; that is to say, of five or six minutes.

* M. Pages, however, the inspecting physician of Bareges, recommends that healthy persons should abstain from the use of mineral waters, as he has seen several who, after three warm baths of three quarters of an hour in length, have been taken with inflammatory fevers which required a vigorous antiphlogistic treatment. In a state of disease, on the contrary, the *tolerance* of the system for the waters is very pronounced; and what is worthy of remark is, that this tolerance diminishes as the disease approaches its cure.

the inspector in administering it, have for their object to produce a general and local excitement, which they ought to graduate according to the nature and degree of the lesion and the temperament of the patient. Thus, for example, in nervous diseases the baths should be of the temperature of the air, and prolonged; the douche should be tepid, short, and in the form of a shower bath; in old rheumatisms, in local paralyses, and in stiffness of joints, recourse should be had to hot baths and douches, and to vapour baths and douches. If it is wished to produce a stimulation still stronger, opposite means are put in play at the same time; thus baths are given at different temperatures, douches given more or less cold in steaming rooms, or cold and hot douches, alternately, to the same individual. Employed under these different forms, mineral waters, when administered by skilful physicians, can be made to fulfil a great number of therapeutical indications.

Though their power of stimulating explains many of the curative effects produced by mineral waters, and though this mode of their action is the most evident and the most easy to prove, still we should be in error were we to suppose that it is the only one. Is it to stimulation alone that can be attributed the efficacy of the waters of Mont d'Or, of Bonnes, and of Canterets, in chronic diseases of the chest? Were this the case all mineral waters would be adapted to the cure of these affections, inasmuch as all are stimulating. If mineral waters acted only as instruments for producing excitement, we can see no reason why a great many other stimulants may not be substituted for them in the treatment of a number of chronic diseases, but which we know are far from being of the same value, and from fulfilling the same indication. It is certain, too, that besides stimulating, mineral waters, according to the difference of their properties, direct their impression specially upon different organs of the economy. Thus sulphurous waters have special action in diseases of the skin, the waters of Vichy in engorgements of the abdominal viscera, the waters of Mont d'Or and Bonnes in chronic diseases of the chest, &c. It is not to be doubted that, conveyed into the circulation, mineral waters have the property of changing the constitution of fluids and solids, of giving consistence or fluidity to the humours; it is for this reason that several writers on therapeutics class them among the alterants. Without being exclusive supporters of the humoral theory, we cannot refuse to admit facts furnished by sound observation; for example, this modification of the humours incontestably belong to the waters of Vichy, for if two glasses of this liquid be taken, containing about two grammes (30.88 grs. Troy) of bicarbonate of soda, the urine becomes promptly alkaline, and a single bath in the same water will produce the same effect; and this alkalization is not limited to the urine; it is remarked, likewise, in the sweat, which in a state of health is always acid. It is probable that all waters which contain a certain quantity of bicarbonate of soda, are endowed with the same property; such are the waters of Vals and of Saint Nectaire.

To sum up, mineral waters act by stimulating our organs, by modifying the humours, and by exercising a specific action upon some one or other apparatus of the economy; these three kinds of action may take place at the same time; for example, the waters of Vichy are exciting, they render all the humours alkaline, and they direct their impression specially upon the viscera placed in the subdiaphragmatic region.

As mineral waters are stimulating to a degree more or less pronounced, it is easy to foresee that they cannot be suitable in acute diseases in which they would augment the activity of the circulation;* they are, on the contrary, of great use in chronic affections, which are aggravated by an active treatment; the good effects arising from their use in such diseases, are the more durable in proportion as they are brought about gradually, or as they are of a chronic nature, like the diseases themselves. Under the influence of mineral waters, the removal of chronic diseases is sometimes effected by critical efforts of nature, which are manifested in the stools, in the perspiration, in the urine, or by the appearance of eruptions on the surface of the body. Sometimes, on the contrary, the cure takes place without any appreciable crisis; the patient experiences no unusual change in his condition; only he continues to get better, and progresses each day towards a cure, without the physician's being able to account for the efficacy of the water. Chalybeate and alkaline waters present particularly this phenomenon.

It is in thermal establishments that it is easy to convince one's self, that many chronic diseases are occasioned and maintained by a suppression of the transpiration of a habitual flux, or of a rheumatismal, gouty, or herpetic principle, which becomes thus thrown back upon the internal organs; but is this retrocession the effect or the cause of the disease? It is evidently the determining cause, when the disorder of the viscus is posterior to the retrocession, and this happens in the greater number of cases. However this may be, it is most rational to endeavour to recall the morbid principle without. Nothing is better adapted for fulfilling this indication than mineral baths, which, in provoking the skin to a moderate congestion of blood, but over a large surface, augment the energy of the cutaneous apparatus, reestablish the important junctions of this emunctory, recall the fluids from the centre to the circumference, and produce often an exanthema resembling scarlatina or a miliary eruption, which is called at Lonesche, *le poussé*. This eruption, brought on by a healthy effort of nature, may produce a revulsion very useful in gastralgias, pulmonary catarrhs of a chronic nature, &c., and is often a precursor of the re-establishment of health.

* The waters of the Seltz, and other cold acidulated waters, are, notwithstanding, sometimes prescribed in typhoid fevers, irritation of the stomach, &c. The bitter saline waters, such as those of Seidlitz and Pullna, are employed in cases of acute diseases, in which most mineral waters would be contraindicated.

We must not think, however, that mineral waters succeed in all chronic diseases; they do injury in lesions of the brain, in epilepsy, apoplexy, aneurisms of the heart and large vessels, in disorganization of the viscera, and in scirrhous and cancerous degenerations; mineral waters, in all these cases, quicken the circulation, excite fever, and hasten the death of the individual.

Mineral waters are so little known as to their therapeutical effects, to the greater number of medical men, that they are often prescribed for patients whom the inspecting physicians are obliged to send away, because they would be more hurtful than advantageous to them. M. Bertrand was forced, in 1836, to advise against the use of the water more than forty patients who had been sent to him.* Similar errors, unfortunately too frequent, bring the medical art into disrepute, and take away all hope from the patient. Persuaded that a portion of these mistakes will be prevented, and that a true service will be rendered to practising physicians, by specifying the cases in which certain mineral waters ought to be preferred, your committee has endeavoured to resolve this problem in therapeutics, by taking for a basis the preceding considerations upon the action of the waters, and principally the facts gathered by the inspecting physicians. We will now communicate to you the remarks which we have made upon this interesting point of practical medicine, in a rapid glance over the chronic diseases, which are met with most frequently at watering places.

* List of diseases for which the use of the waters of Mont d'Or was thought, by M. Bertrand, in 1836, not to be advised.

| | |
|---|---|
| Hypertrophy of the heart, | 4 |
| Phthisis in the last stage, | 9 |
| Active Pneumonhagia, | 5 |
| Scirrhus of the stomach. | 3 |
| Hysteria in a high degree, | 4 |
| Hemiplegia, with cerebral congestion, | 3 |
| Chronic metritis, with fever, | 3 |
| Venereal Periostosis, | 3 |
| Rheumatism, with hectic fever, | 5 |
| Acute gout, | 2 |
| | 41 |

